# Clinical and Basic Research Progress on Treg-Induced Immune Tolerance in Liver Transplantation

**DOI:** 10.3389/fimmu.2021.535012

**Published:** 2021-05-20

**Authors:** Xuhao Ni, Qi Wang, Jian Gu, Ling Lu

**Affiliations:** ^1^ Jiangsu Cancer Hospital, Jiangsu Institute of Cancer Research, Nanjing Medical University Affiliated Cancer Hospital, Nanjing, China; ^2^ Hepatobiliary Center, The First Affiliated Hospital of Nanjing Medical University, Nanjing, China; ^3^ Research Unit of Liver Transplantation and Transplant Immunology, Chinese Academy of Medical Sciences, Nanjing, China; ^4^ Key Laboratory of Liver Transplantation, Chinese Academy of Medical Sciences, NHC Key Laboratory of Living Donor Liver Transplantation, Nanjing, China

**Keywords:** clinical trial, Foxp3, operational tolerance, regulatory T cell, liver transplantation

## Abstract

Rejection after organ transplantation is a cause of graft failure. Effectively reducing rejection and inducing tolerance is a challenge in the field of transplantation immunology. The liver, as an immunologically privileged organ, has high rates of spontaneous and operational tolerance after transplantation, allowing it to maintain its normal function for long periods. Although modern immunosuppression regimens have serious toxicity and side effects, it is very risky to discontinue immunosuppression regimens blindly. A more effective treatment to induce immune tolerance is the most sought-after goal in transplant medicine. Tregs have been shown to play a pivotal role in the regulation of immune balance, and infusion of Tregs can also effectively prevent rejection and cure autoimmune diseases without significant side effects. Given the immune characteristics of the liver, the correct use of Tregs can more effectively induce the occurrence of operational tolerance for liver transplants than for other organ transplants. This review mainly summarizes the latest research advances regarding the characteristics of the hepatic immune microenvironment, operational tolerance, Treg generation *in vitro*, and the application of Tregs in liver transplantation. It is hoped that this review will provide a deeper understanding of Tregs as the most effective treatment to induce and maintain operational tolerance after liver transplantation.

## Introduction

Liver transplantation is effective for end-stage liver disease and acute liver failure, even when used as the sole treatment (1). Over the past few decades, surgical techniques for liver transplantation have matured. Modern immunosuppression regimens have greatly reduced the early mortality of transplant patients. However, diseases caused by the side effects of those regimens also reduce long-term quality of life and increase long-term mortality for recipients, who risk adverse effects such as renal insufficiency and renal failure, cholangitis and bile duct stones caused by biliary tract injury, and tumours caused by immunodeficiency (2). Therefore, exploring more effective and less toxic treatments to induce immune tolerance has become the chief scientific concern in transplantation.

Immune tolerance refers to a specific non-responsive state that the immune system exhibits when exposed to antigenic substances (3). The study of immune tolerance induction has achieved promising results in animal experiments; for example, allografts could maintain good graft function without the use of immunosuppression regimens. For decades, a small number of transplant recipients have shown no signs of rejection and good graft function with long-term discontinuation of immunosuppressants, a phenomenon known as spontaneous operational tolerance (4). In the 1990s, the University of Pittsburgh in the United States found that approximately 20% of liver transplant patients could safely stop all immunosuppressant therapy after the transplant had been in place for many years (5). However, organ damage or failure caused by the side effects of immunosuppressants often occurred early postoperatively and was irreversible. Hence, how to induce early operational tolerance of transplantation through intervention measures is an important research topic at present.

Tregs are a subgroup of immune cells with strong regulatory functions that play an important role in maintaining immune homeostasis and inducing immune tolerance (6). In 1995, Sakaguchi et al. discovered and defined it as a CD4^+^CD25^+^ T cell subset originating from the thymus (7). But Foxp3^+^ T cells were called Tregs when the key transcription factor Foxp3 was discovered in 2003 (8). Current research has clarified that Tregs regulate immune balance mainly by means of direct cell contact and indirect secretion of cytokines (9). Tregs are related to the occurrence of spontaneous immune tolerance after transplantation, and there is a high quantity of Tregs in these patients (10). In recent years, multiple centres have applied *in vitro*-induced Tregs to the induction of early or late tolerance in patients with liver transplantation, and some progress has been achieved (11). This review will systematically summarize the latest research progress and look forward to future research directions.

## The Immunologic Characteristics of Liver

The liver was defined as a non-immune organ in the past and is mainly responsible for the functions of material metabolism, nutrient storage and decomposition of toxic substances. Transformed into continuous understanding of the characteristics of liver tissue, we know that it is also an extremely complex immune organ, with functions such as secreting acute phase proteins, complement components, cytokines and chemokines, and contains a variety of resident immune cells with self-renewal capabilities (11, 12). The liver has been stimulated by a large number of external antigens for a long time because it receives blood from the entire digestive tract, but the liver maintains its autoimmune balance through an extremely complex regulatory network. The recipient immune system is mainly composed of resident immune cells from donors and circulating immune cells from recipients after transplantation. However, the liver, unlike other solid organs, is more likely to coexist with the donor’s immune cell to form immune tolerance, which is inseparable from the internal environment unique to the liver. An explanation may be the presence of chimerism, which is developed by lymphocytes and dendritic cells from donors migrating to the lymph nodes and thymus of recipients, releasing soluble MHC molecules, deleting colonies and exhausting alloreactive T-cells (13). In addition, the portal vein and hepatic artery converge in the hepatic sinus, which results in hypoperfusion pressure, slow blood flow, and a hypoxic state in the sinusoidal area. This provides a favourable place for adaptive immune cells and innate immune cells to contact and respond to each other.

The liver has its own unique innate immune system and plays a key role in the development of immune tolerance after liver transplantation, including liver-derived dendritic cells, Kupffer cells, sinusoidal endothelial cells, natural killer cells, and natural killer T cells (14). A large number of studies have shown that the maturity of dendritic cells in the liver is much lower than that of peripheral lymphoid organs (15–20). Immature dendritic cells display lower expression of MHC-II, costimulatory signalling molecules and IL-12p70 and high expression of IL-10, IL-27 and TGF-β (21–23). Therefore, it is conducive to the expansion of Tregs and the maintenance of their functions but inhibits T cell activation (17, 24–26). Chen et al. recently confirmed that immature dendritic cells overexpressing IL-10 and Fasl display lower expression of MHC-II, CD80 and CD86, which could effectively induce early immune tolerance after liver transplantation in rats (27). Experimental results from our centre showed that galectin-1 induces peripheral monocytes to differentiate into immature dendritic cells, promotes their expression of IL-27 and TGF-β, induces differentiation and expansion of Tregs, and effectively induces immune tolerance after liver transplantation in rats (28). The above conclusions favourably determine the important role of immature liver dendritic cells in the induction of immune tolerance after liver transplantation. Kupffer cells, as the main resident macrophages in the liver, play a critical role in the inflammatory response caused by ischaemia-reperfusion in liver transplantation (29, 30). However, studies have also found that Kupffer cells can induce Tregs to proliferate and secrete IL-10 through direct contact with Tregs while inhibiting T cell activation by secreting PGE2 and 15d-PGJ2 (31–34). Sinusoidal endothelial cells, as the main components of liver non-parenchymal cells, can induce T cell apoptosis by inducing the expression of PD-L1 and inhibiting T cell secretion of IL-2, thereby inducing immune tolerance (35, 36). Meanwhile, studies have shown that hepatic sinusoidal endothelial cells can induce CD4^+^ T cells to differentiate into CD4^+^CD25^low^Foxp3^-^specific T cell subsets with inhibitory activity (37). NK cells have been demonstrated to play dual roles in liver immunity (38). NK cells have been clarified to inhibit dendritic cell activation and promote hepatic tolerance by secreting TGF-β and IL-10, which further induce the expansion of Tregs (39). The above research conclusions suggest that in addition to directly affecting T cells, liver innate immunity can simultaneously induce the differentiation and proliferation of suppressive T cell subsets, especially Tregs.

## Tregs Developments and Foxp3 Regulation

Over the past 20 years, the biological characteristics and immune regulation mechanisms of Tregs have been widely studied. Tregs are classified as thymus-derived Tregs (tTregs) and peripheral-derived Tregs (pTregs) according to the different sites where Tregs differentiate (40, 41). However, tTregs and pTregs are not only different in the place of differentiation but also in the manner of differentiation. tTregs are mainly induced by autoantigens in the thymus, and CD4 single-positive cells express Foxp3 under moderate autoantigen and IL-2 signal stimulation *via* TCR (42, 43). pTregs are mainly induced by foreign antigens, and peripheral CD4^+^ naïve T cells express Foxp3 under the stimulation of bacterial or food antigens and differentiate into pTregs (44, 45). Studies have also confirmed that TCR is essential for the activation, maturation, and functions of Tregs (46, 47). TCR signal activation plays a key role in the differentiation and activation of Tregs and pTregs. Sidwell et al. found that the transcription factor Bach2 inhibits signal transduction downstream of TCR and affects Treg activation. ChIP-seq and ATAC-seq revealed that Bach2 antagonizes TCR-induced IRF4 and DNA binding activity and restricts chromatin accessibility (48). Using single-cell RNA sequencing, Zemmour et al. analysed the variation in TCR expression profiles between Tregs and CD4^+^Foxp3^-^T cells (49). However, there are no reports about alloantigen-reactive Tregs in patients with liver transplantation. Single-cell analysis can provide a deeper understanding of the specificity of TCRs and related transcription factors or key factors and, in combination with ChIP-seq and ATAC-seq, further analyse specific mechanisms. In addition to TCR signalling, TGF-β and IL-2 signalling also play an important role in Treg development, whether in the thymus or in the periphery. Our previous results showed that TGF-β signalling plays a pivotal role in iTreg (induced in cell culture) induction, which mainly depends on downstream SMAD2/3 activation (50). A recent study reported that 5-aza-dC efficiently generates Foxp3+ iTreg TCR-stimulated CD4^+^Foxp3^−^ T cells in the absence of exogenous TGF-β and IL-2, and they further discovered that the function of 5-aza-dC on Treg generation is critically dependent on TGF-βR and IL-2R signalling (51). Although those studies provided us with a deep understanding of the molecular mechanisms underlying the process of Foxp3 induction, we need to look for more drugs or molecules to assist TGF-β and IL-2 in inducing stable iTregs.

The maintenance of the phenotype and function of Tregs depended on the stable expression of Foxp3 and the function of Foxp3 protein. In 2017, we systematically summarized the important regulatory molecular mechanisms affecting Foxp3 at the level of transcription, translation, and post-translational modification (52). The execution of these suppressive functions requires the proper regulation of Foxp3 genes within Treg cells. Many transcription factors can bind to the promoter regions of its gene, such as NAFT, RUNX1, and IRF4 (53–55). Previous data have shown that atRA increases histone acetylation on the Foxp3 gene promoter and CpG demethylation in the region of the Foxp3 locus (56, 57). Our recent research found that YAP upregulates activin receptor expression through binding to TEAD, thereby promoting the activation of the TGF-β/SMAD2/3 signalling pathway, stabilizing and increasing Foxp3 expression and Treg function (58). At the same time, we confirmed for the first time that Foxp3 is regulated by K63-type polyubiquitination. When TRAF6 is defective in Tregs, K63-type polyubiquitination of Foxp3 is significantly inhibited, and its nuclear distribution is significantly abnormal (59). The post-translational modification of Foxp3 has been gradually valued. In addition to ubiquitination, Foxp3 lysine acetylation is also important. Dahiya et al. found that HDAC10 regulates Foxp3 protein stability and transcriptional activity, and HDAC10 deficiency leads to a significant decline in Treg immunosuppressive function (60). Xiao et al. recently found that EZH2 inhibits Foxp3 transcription by downregulating RUNX1 and upregulating SMAD7 expression, further clarifying that methylation modification plays an important role in the regulation of Foxp3 transcription (61). Many studies have deeply determined the molecular mechanism of Foxp3 and other important factors regulating the function of Tregs (62–64). However, the recognition of alloantigen-reactive Tregs is still almost completely unknown. We need to establish an effective system to analyse the regulatory characteristics of alloantigen-reactive Tregs so that we can better and more effectively induce and maintain them and induce stable and durable immune tolerance.

## Tregs and Operational Tolerance

Operational tolerance is different from what we usually call immune tolerance. This means that the allograft does not suffer a rejection reaction and maintains good graft function and normal histology. Because of the unique histological and immune microenvironment characteristics of the liver, it is more prone to spontaneous operational tolerance than other solid and non-solid organs. At first, Starzl found that some patients who discontinued immunosuppressive drugs due to serious side effects did not develop rejection and form natural tolerance (65). Subsequently, Mazariegos recruited 95 liver transplant recipients who had taken immunosuppressive drugs for a long time after operation and had stable liver function to perform withdrawal experiments and found that spontaneous operational tolerance occurred in approximately 20% of recipients (66). The results of clinical withdrawal experiments from multiple centres in the world also confirmed the above conclusions (67–74). The overall incidence of spontaneous operational tolerance in liver transplant recipients remains unknown. Considering that blind withdrawal early can lead to more serious consequences, how to induce early operational tolerance in liver transplant recipients is a major scientific issue in the transplant world today.

Tregs induce immune tolerance through a variety of pathways, including direct and indirect pathways. For example, Tregs interact with B cells, T cells and DCs and inhibit their activation and proliferation by expressing PD-1, CTLA-4, CD39 and LAG-3. It can also secrete the anti-inflammatory cytokines IL-10, IL-25 and TGF-β to inhibit T cell activation, releasing perforin and granzyme to promote target cell apoptosis and competing with T cells for binding to IL-2 by expressing CD25 (75, 76). Th17 cells produce IL-17A, IL-21 and IL-22, which have been shown to promote immunopathology and autoinflammatory diseases (77). Many studies have shown that Tregs suppress Th 17 cell proliferation and control its response (78). Early studies found that the occurrence of acute rejection after liver transplantation was inversely related to the number of peripheral circulating Tregs and the ratio of Tregs/Th17 cells (79–82). Li et al. used a CD25 antibody (250 μg/d, IP) to treat a mouse transplantation model and found that it reduced the proportion of CD4+CD25+ Treg/CD3+ T cells and significantly reduced the incidence of spontaneous tolerance in transplanted mice (83). A clinical trial of withdrawal of recipients who took immunosuppressive drugs after liver transplantation with stable liver function for more than 2 years found that the level of Foxp3 mRNA in peripheral blood of recipients who did not have rejection after withdrawal was increased a rate of 3.5 times each time and continued to increase until the drug was completely withdrawn, but the recipients who experienced a rejection after drug withdrawal could not see this phenomenon (74). Recent studies have used flow cytometry to detect the ratio of Tregs/Th17 in the peripheral blood of patients with rejection within 2 weeks to 1 month after living donor liver transplantation and found that the occurrence of early rejection is directly related to the low number of Tregs (80). Therefore, we can easily predict that Treg immunotherapy may be the most effective way to induce operational tolerance in the early stage.

## Ex Vivo Regulatory T Cells Generation

Since Tregs only account for 5-10% of peripheral blood CD4+ T cells, to obtain a sufficient number of Tregs, we need to expand Tregs *in vitro*. Currently, there are two methods expanding Tregs *in vitro* for clinical applications that are certified by GMP (84). Considering the timeliness of magnetic bead sorting, GMP stipulated that two-step magnetic bead sorting (CliniMACS) is used to obtain human peripheral blood CD4+ CD25+ Tre (85). Treg expansion *in vitro* is mainly divided into polyclonal Treg expansion and alloantigen-reactive Treg expansion. Polyclonal Tregs are expanded by using CD3 and CD28 antibody-coated magnetic beads and IL-2 recombinant protein (86, 87). However, this expansion method inevitably led to the loss of Foxp3 and changed the Treg phenotype, and the effector T cells also expanded and mixed in the presence of IL-2. We and other laboratories added rapamycin and all-trans retinoic acid to effectively maintain Foxp3 expression and inhibit effector T cell expansion (57, 88). Due to the poor specificity of polyclonal Treg antigens, we are now focusing more on alloantigen-reactive Treg expansion. Alloantigen-reactive Tregs can be expanded by using donor antigen-presenting cells, such as dendritic cells, B cells, and peripheral blood mononuclear cells (89). Putnam et al. used CD40L-activated allogeneic B cells for the first time to stimulate and select alloantigen-reactive Tregs and then performed 200-4000 times in 16 days with magnetic beads coated with CD3 and CD28 antibodies and IL-2 recombinant protein (90). Our centre designed a method inducing alloantigen-reactive Tregs and is applying for Republic of South Africa Patents (International Application NO: PCT/CN2018/075730). The invention adopts rapamycin combined with TGF-β cells to induce human T cells into alloantigen-reactive Tregs with immunosuppressive function *in vitro* by the action of DC cells from donors. Podestà et al. used PBMCs to establish an allogeneic mixed lymphocyte system, applied this system to expand alloantigen-reactive Tregs, and added ceprizumab, a CD2 monoclonal antibody. They found that ceprizumab can greatly reduce the proportion of CD4+ and CD8+ effector and memory T cells and at the same time selectively promote alloantigen-reactive Treg expansion (91). This study suggests that we can modify polyclonal Tregs and alloantigen-reactive Tregs *in vitro* based on the biological characteristics of Tregs and the regulatory mechanism of Foxp3 stability so that they have stronger expansion ability and stability.

## The Application of Tregs in Liver Transplantation

As of January 2020, there are very few clinical trials reporting that Tregs successfully induced operational tolerance in patients with liver transplant in the early stage, almost all of which are in phase I/II clinical trials. Ex vivo expanded polyclonal regulatory T‐cell therapy is being utilized in the ThRIL trial at King’s College Hospital, UK [clinicaltrials.gov NCT02166177]. The DeLTA and ARTEMIS trials at University of California, San Francisco, USA, are using donor‐alloantigen‐reactive regulatory T cells (darTregs) [NCT02188719] NCT02474199. A preliminary study from Japan showed that Tregs can safely and effectively induce operational tolerance in the early stage of recipients after living liver transplantation. Treg-enriched allogeneic lymphocytes were obtained by co-culturing recipient spleen lymphocytes and irradiated donor lymphocytes in the presence of CD80 and CD86 antibodies, which were reinfused (23.30 + 14.38×106/kg) on the 13th day after living-donor liver transplantation. Dug withdrawal gradually started after 6 months and completed withdrawal until 18 months. Ten patients were included in this study, and no severe side effects occurred after cell therapy. All patients had normal liver function and liver histology. Seven patients achieved operational tolerance. Three of seven patients resumed taking low-dose immunosuppressive drugs due to autoimmune liver disease. However, this study has no long-term data or follow-up (92). This study suggests that Tregs induce operational tolerance to be safe and effective (**Table 1**).

**Table 1:** The clinical trial for tregs in liver transplantation.

Status	Study Title	Conditions	Trial ID
Active, not recruiting	Safety Study of Using Regulatory T Cells Induce Liver Transplantation Tolerance	Chronic Rejection of Liver Transplant	NCT01624077
Recruiting	Liver Transplantation With Tregs at MGH	Liver Transplantation	NCT03577431
Active, not recruiting	Efficacy of Low Dose, SubQ Interleukin-2 (IL-2) to Expand Endogenous Regulatory T-Cells in Liver Transplant Recipients	Liver Transplantation	NCT02739412
Completed	Donor Alloantigen Reactive Tregs (darTregs) for Calcineurin Inhibitor (CNI) Reduction	Liver Transplant Recipient, Living Donor (of the Respective Liver Transplant Recipient)	NCT02474199
Completed	Safety and Efficacy Study of Regulatory T Cell Therapy in Liver Transplant Patients	End-stage Liver Disease	NCT01678937

## Future Directions

Along with the application of Tregs in inducing operational tolerance after solid organ transplantation and non-solid organ transplantation (93–95), it has been clearly confirmed that Tregs can effectively induce and maintain operational tolerance early without significant side effects. However, although the biological characteristics of Tregs and the molecular regulatory mechanisms of Foxp3 are understood in depth, little is known about the heterogeneity of alloantigen-reactive Tregs in different organs. In the future, it is necessary to further characterize the phenotypic and functional differences in alloantigen-reactive Tregs between different organs *via* modern omics analysis. With that knowledge, we could effectively modify Tregs during *in vitro* expansion to obtain Tregs with stronger suppressive activity and stability and generate common Car-Tregs with antigen-specific properties. For Car-Tregs, it is important to determine and verify the best target for engineered Treg cells, as well as consider whether the target molecule on these cells could be a soluble antigen instead of a surface molecule.

The mass production process of Treg cells is still not perfect, mainly due to the limitations of reagents and equipment. Combining MACS with FACS may further improve this process. The low proliferation rate of Treg cells *in vitro* is in stark contrast to their highly proliferative state *in vivo*. Suitable media, growth factors and stimulants for Treg cells have not been developed. In addition, current Treg cell manufacturing processes are expensive and labour intensive. Maximizing automation not only reduces costs but also improves repeatability and standardization.

Meanwhile, because there is still no effective way to evaluate the outcome of Treg infusion *in vivo*, we need to compare the differences in Treg heterogeneous subgroups *in vivo* before and after Treg therapy and to clarify the phenotypic and functional differences. A better understanding of how Treg cells maintain tissue integrity during homeostasis and in autoimmunity and organ transplantation, whether (and how) Treg cells change their identity in autoimmunity and whether Treg cells from patients with autoimmune disease are intrinsically defective and thus unsuitable for therapeutic use will also be critical to establish a Treg immunotherapy evaluation system that can guide the withdrawal process. In addition to Tregs alone, we need to explore the efficacy of Tregs combined with other immune cell therapies, such as MSCs, DCs or others. In the next few years, as clinical experimental data from other centres are reported, we will achieve a deeper understanding of the efficacy, safety, and side effects of Treg therapy in liver transplantation. However, we still need to establish a safe, effective and unified system to facilitate the implementation of Treg.

## Author Contributions

XN, QW and JG participated in manuscript writing and editing. LL contributed to manuscript editing. All authors contributed to the article and approved the submitted version.

## Funding

This work was supported by grants from the Research Unit of Liver Transplantation and Transplant Immunology of Chinese Academy of Medical Sciences (2019-I2M-5-035), the National Natural Science Foundation of China (81971495, 81571564, 91442117, 81521004, 81522020, 81270553, 81300363, 81772569, and 81572370), and the 863 Young Scientists Special Fund (SS2015AA020932).

## Conflict of Interest

The authors declare that the research was conducted in the absence of any commercial or financial relationships that could be construed as a potential conflict of interest.
